# Multimorbidity before, during and after pregnancy among women in low-income and middle-income countries: protocol for a scoping review

**DOI:** 10.1136/bmjopen-2025-104565

**Published:** 2025-09-23

**Authors:** Vundli Ramokolo, Rifqah A Roomaney, Monique Maqungo, Makandwe Nyirenda, Parul Puri, Kenneth Yakubu, Balaji Gummidi, Wanga Zembe-Mkabile, Xiaolin Xu, Mary McCauley

**Affiliations:** 1 HIV and Other Infectious Disease Research Unit, South African Medical Research Council, Cape Town, Western Cape, South Africa; 2 Burden of Disease Research Unit, South African Medical Research Council, Tygerberg, Western Cape, South Africa; 3 Burden of Disease Research Unit, South African Medical Research Council, Cape Town, Western Cape, South Africa; 4 Burden of Disease Research Unit, South African Medical Research Council, Tygerberg, South Africa; 5 School of Nursing and Public Health, University of KwaZulu-Natal, Durban, KwaZulu-Natal, South Africa; 6 The George Institute for Global Health India, New Delhi, Delhi, India; 7 The George Institute for Global Health, Sydney, New South Wales, Australia; 8 The George Institute for Global Health, Hyderabad, India; 9 Manipal Academy of Higher Education, Manipal, India; 10 Health Systems Research Unit, South African Medical Research Council, Cape Town, Western Cape, South Africa; 11 School of Public Health and School of Medicine, Zhejiang University, Hangzhou, Zhejiang, China; 12 The University of Queensland, Brisbane, Queensland, Australia; 13 Queen Elizabeth Central Hospital, Blantyre, Malawi; 14 University of Liverpool, Liverpool, UK

**Keywords:** pregnancy, multimorbidity, prenatal diagnosis, maternal medicine

## Abstract

**Abstract:**

**Introduction:**

The co-occurrence of multiple long-term conditions, that is, multimorbidity, is increasing globally and is associated with lower quality of life and increased risk of death. The risk and prevalence of multimorbidity are higher among women compared with men, but currently, evidence focusing on women’s multiple long-term conditions during the perinatal period is limited. Existing evidence needs to be examined to determine the extent to which maternal multimorbidity or women’s multiple health needs related to pregnancy have been addressed, especially for women living in low-income and middle-income countries (LMICs) where this burden of disease is the highest. The objective of this scoping review is to map existing evidence in LMICs on (a) Study designs and data sources, (b) Context-relevant definitions and descriptions, (c) Associated risk and protective factors, (d) Relevant maternal and infant health outcomes and (e) Treatments and interventions used to manage multiple long-term conditions before, during and after pregnancy.

**Methods and analysis:**

This scoping review will be conducted using Joanna Briggs Institute methodology and reported according to the Preferred Reporting Items for Systematic Reviews and Meta-Analyses statement extension for scoping reviews. This review will include observational, experimental or quasi-experimental studies, as well as systematic or umbrella reviews, on multimorbidity in women of reproductive age (15–49 years) in prepregnancy, pregnancy or up to 6 weeks after childbirth in LMICs. The studies will focus on definitions, risk and protective factors and management strategies for multiple long-term conditions before, during and after pregnancy. Studies of morbidity in women with a single index condition or conditions that are not related to pregnancy or childbirth will be excluded. A search strategy will be developed using thesaurus (including MeSH) and free-text terms for ‘maternal morbidity’ or ‘multiple long-term conditions’ and associated keywords such as multimorbidity, co-morbidity and unmet health needs related to pregnancy and/or childbirth for women living in LMICs. Electronic (EBSCOhost (CINAHL Ultimate, STM Source, Medline Ultimate), Cochrane Library, Web of Science or Scopus and Google Scholar) and grey literature databases will be searched from database inception. Reference lists and bibliographies of key topic articles will also be searched, and any additional papers that meet the inclusion criteria will be obtained. There will be no limitations on dates or languages. Records will be independently screened, selected and extracted by two researchers. Data will be presented in tables and narrative summaries.

**Ethics and dissemination:**

Ethics approval is not required as this scoping review will summarise previously published data. Findings from the review will be disseminated through various platforms, including peer-reviewed journals, conferences and community meetings.

**Study registration:**

Open Science Framework (https://doi.org/10.17605/OSF.IO/FYCR8).

**STRENGTHS AND LIMITATIONS OF THIS STUDY**

- This scoping review will describe the burden and measurements of multiple long-term conditions and highlight unmet health needs for women living in low-income and middle-income countries before, during and after pregnancy to inform future studies and decision-making around the development of interventions for integrated care.
- The scoping review will be conducted in accordance with the Joanna Briggs Institute methodology, and reported according to the Preferred Reporting Items for Systematic Reviews and Meta-Analyses statement extension for scoping reviews.
- As this is a scoping review, we will not be doing a risk of bias assessment or grading of evidence from the included studies.

## Introduction

 Multimorbidity is a global public health concern that affects millions of people and results in reduced quality of life, increased burden on health systems and increased rates of mortality. Multimorbidity is defined as the co-occurrence of two or more chronic health conditions in an individual and is often referred to using similar terms such as co-morbidities or multiple long-term conditions, or multiple health needs.^1^,^3^ The prevalence of multimorbidity is increasing, and the risk is higher among women compared with men (11, 12). Recent studies highlight that chronic diseases, especially cardiovascular disease, chronic hypertension, obesity and diabetes mellitus, are significant contributors to multimorbidity and often polypharmacy is required to manage the different conditions.^4^,^6^ Although multimorbidity tends to increase with age and is more pronounced in older individuals aged >60 years, recent studies suggest that it is also prevalent among younger populations including women of reproductive age, particularly those living in low-income and middle-income countries (LMICs) where the burden of disease is high.^7 8^ To date, there is limited evidence on multiple long-term conditions affecting women of reproductive age during pregnancy and after childbirth.^4 9 10^

Current data suggest that maternal multimorbidity during pregnancy and after childbirth is rising^3 11^ and is associated with multiple high-risk factors, including those related to socio-demographic and behavioural risks, such as advanced maternal age and substance use during pregnancy, and chronic conditions prior to pregnancy.^12^,^14^ The rise in maternal multimorbidity is concerning, given its strong association with adverse maternal^15^ and perinatal^16 17^ outcomes. Despite the increase in awareness of the co-occurrence of multiple long-term conditions, obstetric research and maternity care in LMICs continue to focus on the effects of individual conditions rather than their combined impact.^18^

The WHO has defined maternal morbidity as ‘any health condition attributed to and/or complicating pregnancy and childbirth that has a negative impact on the woman’s well-being’^19 20^ and has proposed a maternal morbidity matrix as a framework in which to consider women’s multiple health needs during pregnancy and after childbirth.^21^ However, there is currently a lack of consensus regarding the components of maternal multimorbidity, and there is a lack of standardised criteria to measure maternal multimorbidity. Several researchers have attempted to describe and/or measure the burden of maternal multimorbidity using different tools, including indexes that were developed in high-income settings.^22^,^24^ Some studies also suggest a more expanded definition of maternal multimorbidity that includes, in addition to physical health (ie, infectious, medical or obstetric), the co-occurrence of psychological (eg, depression, suicidal ideation) and social (eg, substance abuse and domestic violence) components of ill-health and have used this approach to measure and highlight the significant burden of maternal morbidity across LMICs.^25^ Understanding the burden of maternal multimorbidity aligns with the global tenet that all women have the right to the highest attainable standard of health, including the physical, psychological and social dimensions of well-being;^26 27^ and the international aim to ensure that every woman in every setting has an equal chance to ‘survive and thrive’ before, during and after pregnancy, and that every mother can enjoy a wanted and healthy pregnancy, safe childbirth and full recovery after childbirth.^28 29^ As the awareness of the importance of the screening and diagnosis of maternal multimorbidity increases, there is also a need to improve the management of the multiple morbidities and their medications (polypharmacy), particularly in settings with inadequate healthcare systems where self-medication is common.^30^ Furthermore, how the healthcare system is organised and how it responds to women’s multiple health needs also requires further exploration so context-relevant processes, systems and referral pathways can be developed to ensure that women’s needs are met during pregnancy and after childbirth.

Therefore, we will systematically scope the extent and nature of the evidence of the study designs and data sources, descriptions and definitions for maternal multimorbidity, the prevalence of associated risk factors and related maternal and infant health outcomes, and management options and referral processes in place for women with multimorbidity, during pregnancy and after childbirth living in LMICs. We conducted a preliminary search of EBSCOhost (CINAHL Ultimate, STM Source, Medline Ultimate), Cochrane Library, Web of Science or Scopus and Google Scholar in February 2025 and did not identify similar reviews underway.

## Aim and review questions

### Aim

This scoping review aims to explore the research landscape associated with multimorbidity for living in LMICs before, during and after pregnancy.

### Objectives

The objective of this scoping review is to map existing evidence in LMICs on (a) Data sources and study designs, (b) Context-relevant definitions and descriptions, (c) Associated risk and protective factors, (d) Maternal and infant health outcomes related to maternal multimorbidity and (e) Treatments and interventions used to manage maternal multimorbidity, co-morbidities and/or multiple long-term conditions before, during and after pregnancy. Specifically, we will explore the following questions in the research landscape in LMICs (a) What is the nature of the evidence for maternal multimorbidity in terms of data sources and study designs?, (b) What are the context-relevant definitions and descriptions of maternal multimorbidity?, (c) What are the associated risk and protective factors relating to maternal multimorbidity?, (d) What relevant maternal and infant health outcomes have been measured? and (e) What are the management strategies available for various maternal morbidities, including physical, psychological and social conditions, before, during and after pregnancy?

## Methods and analysis

This scoping review will be conducted in accordance with recommendations outlined by the Joanna Briggs Institute,^31^ and reported according to the Preferred Reporting Items for Systematic Reviews and Meta-Analyses statement extension for scoping reviews (PRISMA-ScR).^22^

### Eligibility

The inclusion and exclusion criteria are summarised in table 1.

**Table 1:** Scoping review inclusion and exclusion criteria

Inclusion	Exclusion
Population	
Studies of multimorbidity in women of reproductive age (15–49 years) before pregnancy, in pregnancy or up to 6 weeks after childbirth	Studies of morbidity in women of reproductive age, focusing on a single index condition
Studies with women with pre-existing conditions prior to pregnancy	Studies of multimorbidity in women that are not related to pregnancy or childbirth
Concept	
Studies focusing on the following: (1) Context-relevant definitions for maternal multimorbidity, (2) Risk and protective factors for maternal multimorbidity and (3) Management strategies for maternal multimorbidity	Studies with no clear definition of maternal multimorbidity or multiple long-term conditions
Context	
Low-income and middle-income countries*	High-income countries*
Primary research studies with observational, experimental and quasi-experimental study designs	Qualitative studies
Systematic reviews and umbrella reviews	Narrative reviews and protocols
Grey literature	

* Note: as defined by World Bank classifications.^34^

### Information sources

This review will focus on research conducted in resource-constrained settings such as LMICs, as defined by World Bank classifications, and thus research focused on high-income countries will be excluded. Our review will be inclusive and will include both primary and secondary research studies on people of diverse racial, religious and ethnic backgrounds. We will include both health facility and community-based studies. We will also not restrict the research studies by the type of morbidities that they report and will include physical, psychological and social components of ill-health.

This scoping review will consider both experimental and quasi-experimental study designs, including randomised controlled trials, non-randomised controlled trials, before and after studies and interrupted time-series studies. In addition, observational studies including prospective and retrospective cohort studies, case-control studies and analytical cross-sectional studies will be considered for inclusion. In addition, relevant systematic or scoping reviews that meet the inclusion criteria will also be considered.

### Search strategy

In consultation with an information specialist, a preliminary strategy was developed and tested in February 2025 using a thesaurus (including MeSH) and free-text terms for concepts of maternal morbidity (eg, maternal multimorbidity, multiple long-term conditions) and pregnancy time frames (eg, pregnancy, prepregnancy, postnatal etc). The keywords will be modified based on words from identified titles, abstracts and index terms (table 2). Electronic databases (EBSCOhost (CINAHL Ultimate, STM Source, Medline Ultimate), Cochrane Library and Web of Science or Scopus) and words from the relevant titles, abstracts and index terms will be used to develop a full search strategy. We will also locate unpublished studies/grey literature from several sources, including Google Scholar, Scopus, websites for conference proceedings, and organisational websites such as WHO. Manual searching will be done in relevant journal portals. The reference list of all included sources of evidence will be screened for additional studies, and authors will be contacted if additional information is required. A preliminary search strategy is shown in table 2 (the search will be updated before completion of the review). There will be no limitations on dates or languages. Non-English language publications will be translated.

**Table 2:** Preliminary search strategy

Database/source	Search terms	Records
PubMed	((((comorbidity(MeSH Terms))) OR (‘multimorbidity’(MeSH Terms))))) OR (‘Multiple long-term conditions’(Title/Abstract) OR ‘Multiple long term conditions’(Title/Abstract))) AND ((humans(Filter)) AND (female(Filter)) AND (adolescent(Filter) OR alladult(Filter)))) AND ((humans(Filter)) AND (female(Filter)) AND (adolescent(Filter) OR alladult(Filter)))) AND ((((‘Pregnancy’(Mesh)))) OR (Postnatal(Title/Abstract)) AND ((humans(Filter)) AND (female(Filter)) AND (adolescent(Filter) OR alladult(Filter))))	1868
Cochrane	#1 MeSH descriptor: (Pregnancy) explode all trees #2 MeSH descriptor: (Comorbidity) explode all trees #3 MeSH descriptor: (Multimorbidity) explode all trees #4 #1 AND #2 OR #3 #5 ‘Multiple long-term conditions’ OR ‘Multiple long-term conditions’ #6 Postnatal #7 #1 OR #6 #8 #2 OR #3 OR #5 #9 #7 AND #8	34 889 5344 172 246 29 7953 40 854 5371 80
Web of Science	#3 ((TI=(multimorbidity)) OR TI=(comorbidity)) OR TS=(‘Multiple long-term conditions’ OR ‘Multiple long term conditions’) and Multimorbidity (OR – Search within topic) and Comorbidity (OR – Search within topic) #6 (TI= (pregnancy and childbirth)) OR ALL=(Postnatal) OR (QMTS=(‘postnatal’)) OR (QMTS=(‘Pregnancy’)) #8 #3 AND #6	136 325 646 634 1545
Google Scholar	Google Scholar(comorbidity OR ‘multimorbidity’ OR ‘Multiple long-term conditions’ OR ‘Multiple long-term conditions’ AND ‘Pregnancy’ OR Postnatal)	500

A preliminary search was conducted on 13 February 2025. The search will be updated before completion of the scoping review. Filters for EPOC low-income and middle-income countries will be applied to the records.

EPOC, Effective Practice and Organisation of Care.

### Study selection

Following the search, all identified citations will be collated and uploaded into EndNote V.20 2020 (Clarivate Analytics, PA, USA) and duplicates removed. Following a pilot test, titles and abstracts will then be screened by two or more independent reviewers for assessment against the inclusion criteria for the review. Potentially relevant sources will be retrieved in full and their citation details imported into the Covidence systematic review software.^32^ The full text of selected citations will be assessed in detail against the inclusion criteria by two or more independent reviewers. Reasons for exclusion of sources of evidence at full text that do not meet the inclusion criteria will be recorded and reported in the scoping review. Any disagreements that arise between the reviewers at each selected stage will be resolved through discussion, or with an additional reviewer/s. The results of the search and the study inclusion process will be reported in full in the final scoping review and presented in a PRISMA-ScR flow diagram.^33^

### Data extraction

Data will be extracted from papers included in the scoping review by two or more independent reviewers using a data extraction tool developed by the reviewers. The data extracted will include specific details about the data sources, participants, concept, definitions, context, time frame of pregnancy, study design and methods, and key findings relevant to the review question/s including what morbidities were measured, how morbidities were defined, what tools were used to measure morbidities, what treatments were used to treat morbidities, what management strategies were applied and gaps in care.

A draft extraction form is provided (box 1). The draft data extraction tool will be modified and revised as necessary during the process of extracting data from each included evidence source. Modifications will be detailed in the scoping review. Any disagreements that arise between the reviewers will be resolved through discussion or with an additional reviewer/s. If appropriate, authors of papers will be contacted to request missing or additional data, where required.

Box 1 **Modified Joanna Briggs Institute data extraction instrument**

**Scoping review details**

Scoping review title
Review objective/s
Review question/s

**Inclusion/exclusion criteria**

Population
Concept
Context
Types of evidence sources

**Evidence source details and characteristics**

Citation details (eg, author/s, date, title, journal, volume, issue, pages)
Country
Context
Participants details (eg, age/sex and number)

**Details/Results extracted from the source of evidence (in relation to the concept of the scoping review**

Maternal multimorbidity definition
Maternal multimorbidity risk factors
Maternal multimorbidity protective factors
Maternal and infant health outcomes related to maternal multimorbidity
Effect measures and/or measures of maternal multimorbidity frequency
Key findings/conclusions

### Data analysis and presentation

**Figure 1:**
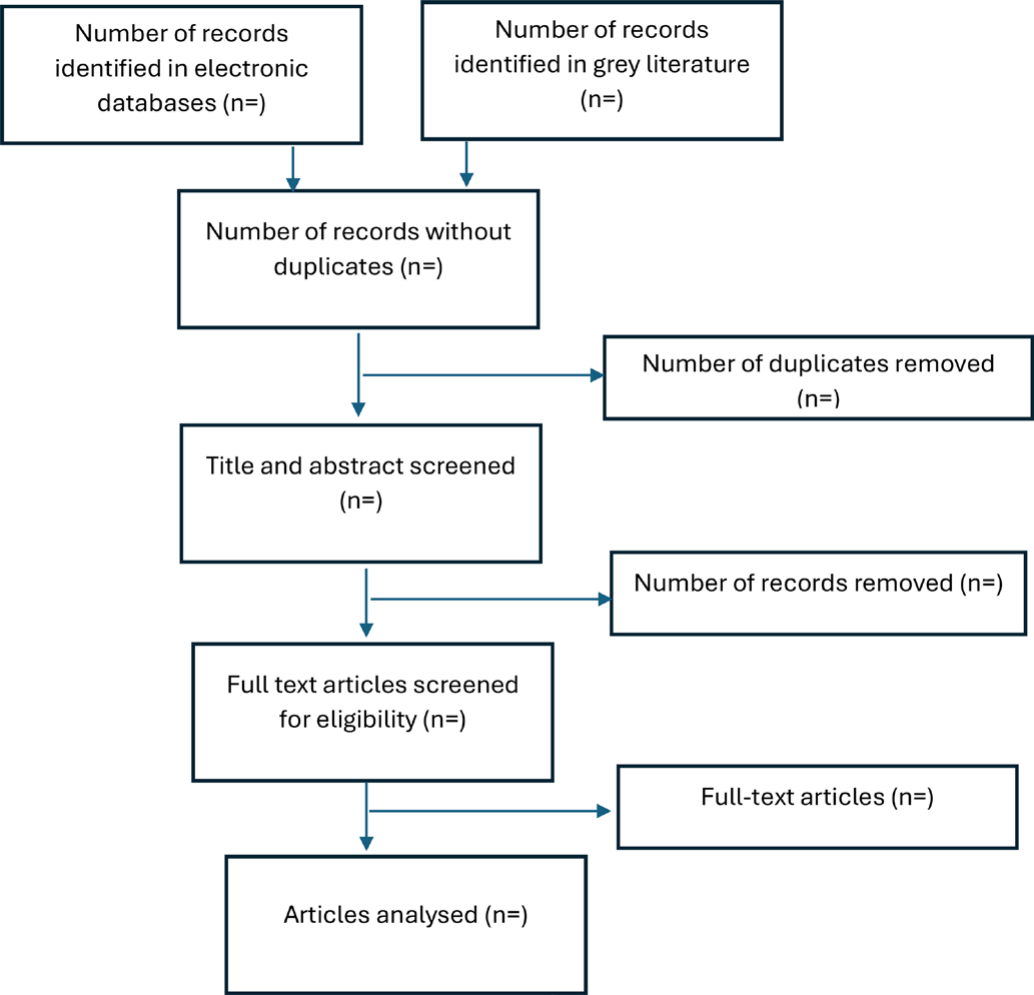
Selection of sources of evidence.

A narrative synthesis of all the included documents will be undertaken according to each research question/objective. Evidence will be presented using tables and diagrams, as appropriate. A flow diagram will depict the selection of studies from the screening until the analysis phase of the review (figure 1). We will note the number of studies on maternal multimorbidity, in which countries the research was conducted and whether the number of articles is increasing with time.

### Patient and public involvement

The scoping review concept, aim and objectives have been shared with community advisory groups to elicit their perspectives. These groups have indicated that the review aim and questions are relevant for their setting and are important to study because of their awareness of large numbers of women who are affected by multimorbidity in their community. They also recommended that the review include pre-conception women, as they would benefit most from preventative interventions for maternal multimorbidity. This protocol was revised accordingly.

### Ethics and dissemination

Ethics approval is not required as this scoping review will summarise previously published data. Findings from the review will be disseminated through various platforms, including peer-reviewed journals, conferences and community meetings.
