# Supplementary material for: Multimorbidity before, during and after pregnancy among women in low-income and middle-income countries: protocol for a scoping review
Source: BMJ Open. 2025 Sep 23;15(9):e104565. doi: 10.1136/bmjopen-2025-104565 (PMC12458865; doi:10.1136/bmjopen-2025-104565)
Supplement: Supplementary file 1 [file bmjopen-2025-104565.reviewer_comments.pdf]

PEER REVIEW HISTORY

BMJ Open publishes all reviews undertaken for accepted manuscripts. Reviewers are asked to complete a checklist review form and are provided with free text boxes to elaborate on their assessment. These free text comments are reproduced below.

ARTICLE DETAILS

Title (Provisional)

Multimorbidity (multiple long-term conditions) before, during and after pregnancy among women in low-income and middle-income countries: protocol for a scoping review

Authors

Ramokolo, Vundli; Roomaney, Rifqah A; Maqungo, Monique; Nyirenda, Makandwe; Puri, Parul; Yakubu , Kenneth; Gummidi, Balaji; Zembe-Mkabile, Wanga; Xu, Xiaolin; McCauley, Mary

VERSION 1 - REVIEW

**Reviewer**	**1**
**Name**	**Santos Rocha, Rita**
**Affiliation**	**Polytechnic Institute of Santarém**
**Date**	**15-Jul-2025**
**COI**	**None**

Very interesting and novel study. Very well-written and clear (although I am not a native English speaker).

Few suggestions to improve the manuscript:

Mention the PRISMA-ScR guideline in the abstract, to clarify the methods.

Add narrative reviews and protocols in the exclusion criteria, as well as umbrella reviews in the inclusion criteria (table 1).

World Bank classifications: reference is missing (website).

Please define "sexual and/or gender minorities". Pregnancy is a female-specific condition; therefore, the search is based on biological sex - women and girls - and not on gender as a social construct.

There will be no limitations on dates or languages. Please explain how many languages will be included. For instance, the Portuguese language is the 5th most spoken language in the world because of Brazil and some countries in Africa.

Conclusions should be replaced by "discussion" section. There are no conclusions on a protocol. The discussion section would benefit from highlighting what is already known on the topic in selected countries.

Implications for practice: This section should be included. For instance, how can the results of this scoping review inform guidelines and policies regarding women's health? Finally, how will this knowledge respond to selected sustainable development goals, namely, No. 3,4,5,17, etc.

VERSION 1 - AUTHOR RESPONSE

Reviewer 1

**Comments/questions**	**Response**
Very interesting and novel study. Very well-written and clear (although I am not a native English speaker).	Thank you for the compliments.
Mention the PRISMA-ScR guideline in the abstract, to clarify the methods.	Thank you for the suggestion. We have added PRIMSA and JBI in the abstract (page 2).
Add narrative reviews and protocols in the exclusion criteria, as well as umbrella reviews in the inclusion criteria (table 1).	Thank you for the suggestion. We have made the suggested changes to Table 1 (page 8)
World Bank classifications: reference is missing (website).	We have added the missing reference (page 8).
Please define "sexual and/or gender minorities". Pregnancy is a female-specific condition; therefore, the search is based on biological sex - women and girls - and not on gender as a social construct.	We agree that pregnancy only pertains to females and therefore removed the statement.
There will be no limitations on dates or languages. Please explain how many languages will be included. For instance, the Portuguese language is the 5th most spoken language in the	We will not restrict the languages (page 9).

world because of Brazil and some countries in Africa.	
Conclusions should be replaced by "discussion" section. There are no conclusions on a protocol. The discussion section would benefit from highlighting what is already known on the topic in selected countries.<br>Implications for practice: This section should be included [within the Discussion section]. For instance, how can the results of this scoping review inform guidelines and policies regarding women's health? Finally, how will this knowledge respond to selected sustainable development goals, namely, No. 3,4,5,17, etc.	Based on feedback from the editor and reviewer we have incorporated the text that was in the 'Conclusions' into the 'Strengths and limitations' section of the scoping review. As such we have decided to forgo the optional 'Discussion' section.
** **<br>COI statement(s):<br><br>Reviewer: 1<br>If you have selected 'Yes' above, please provide details of any competing interests.: Not applicable.	No conflict of interest declared
	We have replaced Ryyan with Covidence systematic review program (pages 10 and 16)
	Dr Gummidi joined the project intermittently. He has now left the project and organization and will not be involved in the scoping review. We have therefore removed him from the author list.
